# Writing self‐efficacy in nursing students: The influence of a discipline‐specific writing environment

**DOI:** 10.1002/nop2.90

**Published:** 2017-07-27

**Authors:** Kim M. Mitchell, Tom Harrigan, Diana E. McMillan

**Affiliations:** ^1^ Nursing Department School of Health Sciences and Community Services Red River College Winnipeg MB Canada; ^2^ College of Nursing Rady Faculty of Health Sciences University of Manitoba Winnipeg MB Canada

**Keywords:** self‐efficacy, undergraduate students, writing, writing anxiety, writing pedagogy, writing scaffold

## Abstract

**Aim:**

To explore if writing self‐efficacy improved among first‐year nursing students in the context of discipline‐specific writing. The relationship between writing self‐efficacy, anxiety and student grades are also explored with respect to various learner characteristics such as postsecondary experience, writing history, English as a second language status and online versus classroom instruction.

**Design:**

A one group quasi‐experimental study with a time control period.

**Method:**

Data was collected over the 2013–2014 academic year at orientation, start of writing course and end of writing course.

**Results:**

Writing self‐efficacy improved from pre‐ to post writing course but remained stable during the time control period. Anxiety was negatively related to writing self‐efficacy but remained stable across the study period. Inexperienced students and students with less writing experience, appeared to over‐inflate their self‐assessed writing self‐efficacy early in the programme. This study gives promising evidence that online and classroom delivery of instruction are both feasible for introducing discipline specific writing.

## 1 INTRODUCTION

Self‐efficacy is a concept that has received an enormous amount of attention since the development of Bandura's theory (1977) but exploring self‐efficacy from the perspective of writing has received sparse consideration. Self‐beliefs are thought to predict academic success and influence career choices and self‐efficacy may be a better predictor of performance than actual ability (Pajares & Valiante, 2006). In nursing, scholars are only beginning to discuss the role of writing self‐efficacy and its relation to student success (Miller, Russell, Cheng, & Skarbek, 2015; Mitchell, Harrigan, Stefansson, & Setlack, 2017) and more research is required to inform this important educational discussion.

Writing self‐efficacy (WSE) can be defined as a writer's belief about their ability to write in a specific context. Bandura's self‐efficacy theory, emphasizes that context critically influences self‐efficacy perceptions (Bandura, 1997). Discipline‐specific writing instruction has been acknowledged as the preferred method for introducing students to nursing's unique discourse through allowing opportunities to practice higher level thinking strategies such as critical analysis. (Andre & Graves, 2013; Luthy, Peterson, Lassetter, & Callister, 2009; Oermann et al., 2014) However, empirical testing of this relationship is lacking likely because so few discipline‐specific writing courses are offered in nursing curriculums. Andre and Graves (2013), who investigated the nature of writing instruction in nursing programmes in Canada, identified that only 6% of programmes included a discipline‐specific course. Close to half of programmes had no required writing course and the remainder required a generic writing course, an English literature course, or both. Thus, research evidence is required to establish the benefits of discipline‐specific writing.

The purpose of the present investigation was to explore, via quasi‐experimental methods, if WSE improved among first‐year nursing students in the context of discipline‐specific writing in one college baccalaureate nursing programme. The relationship between WSE, anxiety and student grades are also explored with respect to various learner characteristics including, past postsecondary educational experience, writing history, English as a second language (ESL) status and online versus classroom instructional environment.

## 2 BACKGROUND

Nursing student populations have been the focus of inquiry in two studies: Miller et al. (2015), who explored writing in a cohort of post‐RN students near the end of their programme in a discipline‐specific environment and Mitchell et al. (2017), who described WSE and anxiety in first‐year nursing students pre and post a discipline‐specific writing course. Thus, given the limited research in nursing on discipline‐specific writing, this literature review will take the approach of an interdisciplinary examination of writing self‐efficacy as it relates to writing performance assessment, improvement in WSE pre‐ to post intervention or course specific strategy and the relationship between WSE and anxiety.

### 2.1 Writing performance

WSE's relationship with writing performance has received attention from various researchers as it correlates with or predicts student grades. A variety of writing activities have been used to define writing performance including on‐demand essays of 30‐minutes or less (Pajares & Johnson, 1994; Shell, Murphy, & Bruning, 1989; Woody et al., 2014), final grades or grade point average (Goodman & Cirka, 2009; Martinez, Kock, & Cass, 2011; Williams & Takaku, 2011; Zimmerman & Bandura, 1994), computer scored essays (Jones, 2008), scaffolded writing assignments and/or papers across a term (Miller et al., 2015; Woodrow, 2011) and complete essays (MacArthur, Philippakos, & Graham, 2016; Mitchell et al., 2017; Prat‐Sala & Redford, 2012; Sanders‐Reio, Alexander, Reio, & Newmann, 2014).

WSE, using various measurement instruments, has only partially been able to predict writing performance in most studies, usually predicting less than 10% of variance in grades (Prat‐Sala & Redford, 2012; Shell et al., 1989), with some studies finding a negligible relationship (Jones, 2008; MacArthur et al., 2016) and only one researcher identifying a “powerful effect” in a population of Chinese students learning to write in English (Woodrow, 2011). Differences in findings may be due to inconsistent methods of assessing performance or to the remote proximity of the WSE measurement to the evaluation of the writing outcome (Pajares & Johnson, 1994).

The study by Williams and Takaku (2011) compared ESL and non‐ESL students in terms of help seeking and writing performance. These authors found that ESL students scored lower than domestic students at the beginning of their freshman year but eventually outperformed domestic students by the end of their sophomore year. Higher self‐efficacy predicted writing centre use and writing centre use became the mediator between WSE and the grade the student ultimately achieved.

### 2.2 Writing self‐efficacy improvement from pre‐ to postwriting course

Among researchers who assessed change in WSE from pre‐ to postwriting course (Goodman & Cirka, 2009; Jones, 2008; MacArthur et al., 2016; Miller et al., 2015; Mitchell et al., 2017; Van de Poel & Gasiorek, 2012; Woody et al., 2014; Xu, Park, & Baek, 2011) all identified a statistically significant improvement in WSE. Given that each of these studies explored different populations using a variety of instructional strategies and different instruments to measure WSE, the consistency of this finding is evidence that WSE can be successfully influenced with positive instruction. Mitchell et al. (2017), providing a course design example, described the role of the instructor in the scaffolding process as including but not limited to anxiety control, simplifying the task demand, providing feedback, ensuring students stay on task and on the specific focus of the assignment, reviewing outlines or drafts of papers and providing models of successful student past writing efforts for current students to follow. Literature describing scaffolding as an instructional model also emphasizes the importance of an instructor “stance” that supports a collaborative instructor–student relationship (Benko, 2013).

### 2.3 The relationship between writing self‐efficacy and anxiety

One instructor role in writing instruction would be to normalize and alleviate writing anxiety. Writing anxiety is a common emotional response to writing tasks no matter the experience level of the writer (Zimmerman & Bandura, 1994). Anxiety, assessed using various measures (Martinez et al., 2011; Mitchell et al., 2017; Woodrow, 2010) and writing apprehension, assessed using Daly and Miller's writing apprehension scale (Goodman & Cirka, 2009; Pajares & Johnson, 1994; Sanders‐Reio et al., 2014) were the most common methods of assessing the emotional arousal component of self‐efficacy theory. Pajares and Johnson described writing apprehension as a form of writing anxiety. Correlations between apprehension or anxiety and WSE are consistently negative regardless of measurement tool used (Martinez et al., 2011; Mitchell et al., 2017; Pajares & Johnson, 1994; Sanders‐Reio et al., 2014). Through use of statistical modelling, researchers have demonstrated that anxiety's role in influencing academic performance is mediated through self‐efficacy (Goodman & Cirka, 2009; Martinez et al., 2011; Woodrow, 2011). Evaluation of change in apprehension from pre‐ to post writing course has been inconsistent with some authors identifying a statistically significant improvement in apprehension in pre‐ to post course methods (Goodman & Cirka) and others finding that apprehension remains constant even when self‐efficacy shows improvement (Pajares & Johnson).

## 3 THE STUDY

### 3.1 Participants

Participants in this study either directly enrolled in the Baccalaureate nursing programme (minimum entry requirement: 60% average in prerequisite courses) or entered through a college preparation programme designed to help mature students update their educational prerequisites. All 192 students registered in three sections of the required course “Scholarly Writing” were eligible to participate. Course sections were offered in both the first and second term of the 2013–2014 academic year. From 192 students, 132 participants (68.8%) provided useable data for analysis. Of the participating students, 27 (20.5%) were enrolled in an online‐only section offered in the first term of the nursing programme, 35 (26.5%) were in an online section in the second term of the programme and 70 (53.0%) were in a second term classroom section.

From the original 192 possible participants, 60 students failed to return sufficient questionnaires (31.2%). An assessment of paper percent grade, using an independent *t* test, comparing participants (mean = 69.85, SD = 18.10) to non‐participants (mean = 56.71, SD 23.88) found significantly lower grades in non‐participants*, t*(187) = 3.72, *p *<* *.001. A similar significant difference was observed between participants (mean = 5.85, SD 12.97) and non‐participants (mean = 64.48, SD = 19.40) on final percentage grade, *t*(187) = 4.05, *p *<* *.001.

### 3.2 Design

**Figure 1:**
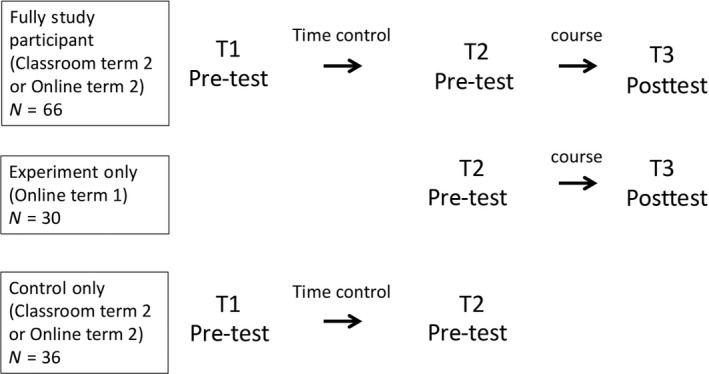
Study groups and context of participation over three measurement time points. Data was collected at T1 (T2 for online term 1 participants) during their first year orientation. T2 data was delivered and collected during the first classroom session for the classroom group and by email for the online group. T3 questionnaires were delivered and returned by email for all groups

The study employed a one‐group quasi‐experimental pre‐test/posttest design with a time‐control period in the term prior to participation. A time‐control was added to rule out possible changes in WSE and anxiety unrelated to participating in a writing course. Figure 1 outlines the study groups that emerged given the varying degrees of participation of students in the context of collecting data over two academic terms in various course sections. Because a time‐control period was not possible for the students registered in the online section offered in the first term of the programme, these students were included as “experiment‐only” participants (*n *=* *30)—three additional participants in this group were students in the other sections who did not return the first questionnaire.

### 3.3 Ethical approval and study procedures

Ethical approval was obtained from the Research Ethics Board at the instructional institution. Informed consent was secured by presenting all participants with a letter attached to the front of a questionnaire package following a presentation about the study during their first‐year orientation (T1). Return of questionnaire was considered consent to participate. Bonus marks, amounting to 2% of the final grade, were offered to students as a reward for participation to help reduce attrition. The pre‐course questionnaire (T2) was distributed on the first day of class for the second term online section and requested by email from the second term online students.

The post course questionnaire (T3) was requested via email from all students following completion of the academic paper assignment but prior to grades being released. This timing was chosen to avoid having student's knowledge of grading and feedback as an influencing factor on their internalized perception of their WSE. Students who did not return the post course questionnaire were included as “time‐control only” participants (*n *=* *36). The main analysis was performed on students who completed all three questionnaires, referred to as “full participants” (*n *=* *66). Online or classroom course section enrolment was self‐selected, however, students in the second term groups were given the general advice to choose the online section only if they felt confident with their writing skills. Students in the first term online section were required to have completed course credits in at least two other first term courses as this section was only added to decrease the class size for second term.

#### 3.3.1 Scholarly writing course description and environment

The course was developed using scaffolding strategies (Benko, 2013; Gazza & Hunker, 2012; Vanderburg, 2006). A complete description of the course learning outcomes and instructional strategies has been presented elsewhere (Mitchell et al., 2017). The main outcome of the course required students to produce a final academic paper worth 60% of their final grade. The instructor provided three to five topic choices. First term students chose from topics such as binge drinking, victim blaming and energy drinks. The second term groups chose from narcotics addiction in health care workers, immunizations, kids and electronic devices, cyber bullying and healthy relationships.

### 3.4 Measures

#### 3.4.1 Self‐efficacy scale for academic writing

The Self‐Efficacy Scale for Academic Writing (SESAW) was designed by the first author of this study. The SESAW is a 10‐item 4‐point Likert scale with response options ranging from strongly agree to strongly disagree. Scale items are presented in Mitchell et al. (2017). Cronbach's alpha for the SESAW was assessed as .82–.85 for this study and .85–.90 for a previous cohort. Concurrent comparisons with the General Self‐Efficacy Scale (Schwarzer & Jerusalem, 1995) were .50 and .53 at pre‐test and posttest, respectively, in previous use.

#### 3.4.2 State‐trait anxiety inventory

The STAI is a two‐part questionnaire measuring anxiety as a stable personality trait (trait anxiety) and as a result of transitory anxiety producing circumstances (state anxiety) (Spielberger, 1983). The STAI has demonstrated acceptable reliability and validity in nursing and education populations. In college students, the test‐retest scores for the trait scale are reported to be .73–.86 but proved less stable in the state scale ranging from .16–.62, which, according to Spielberger, was expected because the state scale should be sensitive to situational factors on a given day. Cronbach's alpha is reported at greater than 0.90 in both scales in a college student population (Spielberger).

#### 3.4.3 Grammar and APA knowledge test

This researcher designed knowledge test was created to assess improvement in recognition of errors in grammar and American Psychological Association (APA) style guide usage. The 10 questions were formatted as written statements that contained one error, or no errors. Errors in punctuation and spelling were not included. Error types included: use of & rather than “and” in an unbracketed APA citation with two authors; two examples where no page number or paragraph number was included with a direct quotation; a then/than grammar error; a sentence fragment; a date separated from its author in citation; two examples involving a word misuse of your/you're or its/it's; and two correct statements. Risk taking was rewarded in the scoring strategy. An unanswered question scored 0, a correct answer scored 2 and an incorrect or partially correct answer scored 1, for a total possible test score of 20. Participants were not informed of the scoring process for this knowledge test.

#### 3.4.4 Assessment of writing

The two course instructors graded the scholarly paper assignments in the first term students. The second term sections required seven graders, including the two course instructors. The department chair assigned five additional markers, who were also classified as instructors in the nursing programme, to assist with the process. These five graders were all provided with the same training by the first author as to how to use the rubric found in Table 1. A printed instruction sheet was supplied to all graders to help ensure consistency. The two course instructors (including the first author) were available for consultation during the process of grading as required and the first author completed a spot check of each external grader to assess for consistency.

**Table 1:** Rubric for grading scholarly paper assignment^a^

Criteria	Exceptional	Meets expectations	Emerging/inconsistent	Poor/absent
Introduction	(Score 3) Clear purpose statement introduces points of analysis. Provides clear point of interest outlining significance/importance of topic. Introduction is an interesting read and makes marker want to learn more about the topic presented	(Score 2–2.5) Purpose statement clear. May lack clear point of interest outlining significance of topic.	(Score 0.5–1.5) Lacks clear purpose statement. Wordy or scant in expression hampering clarity.	(Score 0) Absent or unrelated to topic
Content expression	(Score 17–21) Clear and consistent synthesis of data. Scholarly voice consistently used. Content depth at high level and paper is an engaging and interesting read.	(Score 13–16.5) Organized, detailed and usually supported with rationale. Synthesis of data at beginning level. Depth of analysis is usually what is missing.	(Score 3.5–12.5) Disorganized. Minimal evidence of data synthesis. Some headings not defined. Lack of rationale for argument or no clear argument. Poor connection between information in paper and main topic. Paper is confusing.	(Score 0–3) Unrelated or only vaguely related to assigned topic.
Research depth	(Score 3.5–4) Goes above and beyond in research expectations and number of sources cited and integrated. Articles are all current (<5 years old) or not inappropriately “old” depending on subject matter. Emphasis is on peer reviewed journals in the writing of the paper.	(Score 2–3) Meets minimum source requirement. May have a few sources above minimum but the extra sources are not high quality sources. (e.g. extra websites). Sources are current and not inappropriately old.	(Score 0.5–1.5) Meets minimum source requirements but use of sources in body of paper emphasize or are heavily weighted to one source or a weaker source type (e.g. websites vs. peer reviewed journals). Most sources are current.	(Score 0) Does not meet requirements for minimum expected sources. Sources old and outdated.
Content mechanics and revisions	Assessment criteria: grammar, typo, punctuation errors minimal (forgive 1 error) Avoids jargon, slang, colloquialisms. Minimal spelling errors (forgive 1 error). Avoids wordiness, redundancy, repetitiveness. Paragraphs divided appropriately. No evidence of awkward, run‐on, fragment sentences. Direct quotes only used for definitions and in other situations where it is critical meaning of original is maintained. Paper length appropriate and per assignment guidelines: ± half page from page limit.			Paper is assessed as having an evaluation of “No” from the list for: 0–1 item (score 8–9) 2–4 items (score 5–7) 5–6 items (score 3–4) 7‐9 items (score 0‐2)
Conclusion	(Score 3) Summarizes paper using a style that synthesizes the main argument/thesis. Conclusion is an interesting read and doesn't feel repetitive but still summarizes the elements of the paper.	(Score 2–2.5) Clearly written. Attempt to not just relist headings. Conclusion might seem as if new ideas are being introduced.	(Score 0.5–1.5) Wordy or scant. Introduces new ideas. Simply relists headings to sum up paper.	(Score 0) Absent or does not summarize the paper written.
APA Marking Guide Score (transferred)	Score 18–20	Score 14–17	Score:10–13	Score 0–9
Overall Score	A or A+ 48–60	B or B+ 42–47	C or C+ 36–41	D or F 0–35

a Developed by K. M. Mitchell (first author).

### 3.5 Analysis

Data was analyzed using the Statistical Package for the Social Sciences version 22.0. Statistical tests performed are identified in the results section. Power was calculated for the main effect of change in self‐efficacy in the full‐participant group using an effect size of .5 and alpha of .05. For this study a sample size of 63 would yield a power of greater than 98%.

## 4 RESULTS

The demographic characteristics of the sample are presented in Table 2 for the total sample and by study group. One‐way Analysis of Variance (ANOVA) failed to identify any statistically significant demographic differences based on study participation level other than the experimental‐only group was significantly younger (*p *=* *.042). Table 3 describes the sample by mean grades, SESAW, APA and grammar score and STAI scores at all three time points. No statistically significant differences were noted between the three study groups, using *t* tests or ANOVA, except in final course percentage grade where the time‐control only group achieved the lowest final grades.

**Table 2:** Demographic characteristics of the sample by participation group

Sample and size^a^	All participants *N *=* *134^b^	Time control only *n *=* *36	Experimental only *n *=* *30	Full participant *n *=* *67
	*N*(%)	*n*(%)	*n*(%)	*n*(%)
Course section				
Online term 1	27 (20.3%)	–	27 (90%)	–
Online term 2	35 (26.3%)	10 (27.8%)	–	25 (37.3%)
Classroom term 2	71 (53.4%)	26 (72.2%)	3 (10%)	42 (62.7%)
Age				
18–24	53 (39.6%)	17 (44.7%)	15 (50%)	21 (31.3%)
25–29	38 (28.4%)	10 (26.3%)	12 (40%)	16 (23.9%)
30–34	20 (14.9%)	5 (13.2%)	2 (6.7%)	13 (19.4%)
35–39	10 (7.5%)	1 (2.6%)	0	9 (13.4%)
40–44	8 (6.0%)	4 (10.5%)	0	4 (6.0%)
45–49	2 (1.5%)	1 (2.6%)	0	1 (1.5%)
50+	3 (2.2%)	0	1 (3.3%)	2 (3.0%)
Gender				
Female	119 (88.8%)	32 (84.2%)	26 (86.7%)	61 (91.0%)
Male	15 (11.2%)	5 (13.2%)	4 (13.3%)	6 (9.0%)
ESL				
Yes	32 (23.9%)	7 (18.9%)	8 (26.7%)	17 (25.4%)
No	102 (76.1%)	30 (81.1%)	21 (70%)	50 (74.6%)
Education				
High school grad	16 (11.9%)	7 (18.9%)	2 (6.7%)	7 (10.4%)
Some college/university	74 (55.2%)	19 (51.3%)	20 (66.7%)	35 (52.2%)
Completed diploma or degree	44 (32.8%)	11 (29.8%)	8 (26.7%)	25 (37.3%)
Writing history				
Never written a paper	22 (18.6%)	7 (20.6%)	3 (12.0%)	12 (20.3%)
Greater than 5 years ago	20 (16.9%)	5 (14.7%)	4 (16.0%)	11 (18.6%)
Less than 5 years ago	69 (58.5%)	19 (55.9%)	18 (72.0%)	32 (54.2%)
Write formally	1 (0.8%)	1 (2.9%)	0	0
Previous writing course credit	6 (5.1%)	2 (5.9%)	0	4 (6.8%)
Paper grade %(range)	20.8%–100%^c^	20.8%–100%	44.5%–94.3%	31%–100%
Final grade %(range)	17.3%–98.3%^c^	17.3%–98.2%	63.0%–95.0%	45.8%–98.3%

a Numbers may not match reported sample sizes due to areas of missing data for some participants.

b Includes some participants who returned questionnaires with incomplete data for main study variables.

c Represents grade ranges for the entire class *N *=* *192 (includes non‐participants).

**Table 3:** Grades, SESAW, APA and grammar and STAI scores by participation group at all time points

	Full participants *n *=* *66 M(SD)	Experiment only *n *=* *30 M(SD)	Time control only *n *=* *36 M(SD)	*F/t*	*p*
Final %^a^	77.33 (11.50)	79.79 (9.56)	69.63 (16.24)	6.31	<0.001
Paper %	71.36 (16.31)	73.44 (14.30)	63.88 (22.76)	2.80	0.06
SESAW T1	26.60 (7.48)	–	29.03 (3.78)	1.848	0.07
SESAW T2	28.57 (3.67)	27.07 (8.46)	28.73 (3.75)	1.08	.34
SESAW T3	30.76 (3.60)	29.77 (3.96)	–	1.218	.23
APA/G T1	9.98 (3.94)	–	10.37 (3.43)	.456	.65
APA/G T2	11.43 (3.29)	10.77 (3.17)	11.11 (3.86)	.363	.70
APA/G T3	14.80 (2.57)	14.33 (3.53)	–	.735	.46
State Anx T1	41.93 (9.96)	–	42.76 (13.11)	.132	.72
State Anx T2	41.36 (9.77)	41.86 (8.90)	42.81 (10.61)	.260	.77
State Anx T3	41.59 (10.61)	41.17 (10.45)	–	.033	.86
Trait Anx T1	39.56 (9.84)	–	38.59 (10.21)	.219	.64
Trait Anx T2	40.78 (8.94)	40.17 (8.35)	39.84 (8.97)	.147	.86
Trait Anx T3	40.28 (10.20)	40.55 (9.19)	–	.015	.90

a Post hoc Tukey Final % Control vs. Full *p *=* *0.01; Post hoc Tukey Final % Control vs. Exp. *p *=* *0.004.

### 4.1 Change over time

Table 4 presents the results of the time effect on the key study variables using repeated measures ANOVA or dependent *t* tests. In the full participant group, as expected, SESAW scores were statistically non‐significant in the control period but significantly improved from pre‐ to post course (*p *<* *0.001). SESAW scores did not achieve statistical significance in either the control‐only participants or the experiment‐only participants during the study period. There was no change in either state or trait anxiety at any time period in any of the three groups. APA and grammar knowledge improved significantly and were different at all three time periods in full participants (*p *<* *0.001) and also improved in the time control‐only group (*p *=* *0.03) and the experimental‐only group (*p *<* *0.001).

**Table 4:** Change in SESAW, APA and grammar knowledge and STAI scores across three time points by participation group

	T1 M(SD)	T2 M(SD)	T3 M(SD)	*F/t*	*p*
Control‐only *N *=* *36					
SESAW	29.16 (3.81)	28.71 (3.70)	–	*t *=* *0.802	ns
APA/G	10.37 (3.43)	11.83 (3.32)	–	*t *= −2.22	0.03
State anxiety	42.76 (13.11)	42.81 (10.61)	–	*t *= −0.028	ns
Trait anxiety	38.59 (10.21)	39.84 (8.97)	–	*t *= −1.01	ns
Experiment‐only *n *=* *30					
SESAW	–	27.07 (8.46)	29.77 (3.96)	*t *= −1.93	0.06
APA/G	–	10.77 (3.16)	14.54 (3.66)	*t *= −4.35	<0.001
State anxiety	–	41.86 (10.41)	41.86 (10.41)	*t *=* *0.00	ns
Trait anxiety	–	40.21 (8.49)	40.55 (9.19)	*t *= −0.396	ns
Full‐participant *n *=* *66					
SESAW *n *=* *63	28.29 (3.32)	28.49 (3.61)	30.81 (3.54)	*F *=* *16.20	<0.001^a^
APA/G *n *=* *60	10.02	11.63	14.85	*F *=* *32.81	<0.001^b^
State anxiety *n *=* *66	41.93 (9.96)	41.36 (9.77)	41.59 (10.61)	*F *=* *0.120	ns
Trait anxiety *n *=* *66	39.56 (9.84)	40.78 (8.94)	40.28 (10.20)	*F *=* *0.506	ns

a Pairwise Comparisons Post Hoc: SESAW T1 and T2 *p *=* *.67; T1 and T3 *p *= <0.001; T2 and T3 *p *= <0.001.

b Pairwise Comparisons Post Hoc: APA/G T1 and T2 *p *=* *0.02; T1 and T3 *p *= <0.001; T2 and T3 *p *= <0.001.

### 4.2 Correlations between SESAW and anxiety

Pearson's r calculated negative correlations, as expected, between SESAW and both state and trait anxiety with the exception of state anxiety at T3. Using Pearson's *r*, correlations with SESAW at T1 were *r *= −.33 (*p *<* *.001) for state anxiety and *r *= −.24 (*p *<* *0.05) for trait anxiety. At T2, SESAW negatively correlated with state anxiety, *r *= −.48 (*p *<* *.001) and trait anxiety, *r *= −.53 (*p *<* *0.001) and at T3 these negative relationships were maintained with state, *r *= −.17 (n.s.) and trait, *r *= −.23 (*p *<* *0.05).

### 4.3 Correlations between SESAW and paper and final percentage grades

As expected, using Pearson's r, both paper and final percentage grades were uncorrelated with SESAW measures more remote from their writing performance at T1 (paper percent *r *= −0.004, *p *= n.s.; final grade percent *r *=* *0.04, *p *= n.s.) and T2 (paper percent *r *=* *0.04, *p *= n.s.; final grade percent *r *=* *0.07, *p *= n.s.) but achieved a statistically significant correlation at the SESAW measure most proximal to their performance at T3 (paper percent *r *=* *.24, *p *<* *0.05; final grade percent *r *=* *.25, *p *<* *0.05).

### 4.4 Online versus classroom instruction

The study hypothesis predicted no differences between the online and classroom experience in terms of grades, APA and grammar test scores and STAI and this hypothesis was observed in the data with the notable exception of final course grade. Using independent *t* tests, final percentage grade differences between the first term online section (mean* *= 80.66, SD = 9.33), the second term online section (mean = 72.84, SD 13.09) and the second term classroom section (mean = 75.49, SD 13.73) grades were non‐significant by ANOVA, *F* (2,129) = 2.90, *p *=* *0.058, but post hoc tests identified a significant difference between the two online groups (*p *=* *0.048). The SESAW was expected to be higher in the second term section of online students because students were guided to choose this section based on their self‐assessed confidence in writing. As expected, independent *t* tests showed SESAW at T1 to differ between the second term classroom (mean = 27.95, *SD* 3.44) and online sections (mean = 29.52, SD 3.26), *t* (96) = −2.16, *p *=* *0.033.

### 4.5 Education prior to nursing admission

Students entering the programme with only high school entry credits fared the worst in terms of their paper and final percentage grades but did not correspondingly differ on SESAW, APA and grammar test, or the STAI scores. This was an expected finding. ANOVA compared the paper percentage grades in high school entry students (mean = 59.83, SD 23.45) with students with previous degrees or diplomas (mean = 74.98, SD 16.79) as well as students with some postsecondary experience (mean = 69.05, SD 16.78) *F* (2, 128) = 4.36, *p *=* *0.015. This pattern mostly held when examining final percentage grades where high school entry students (mean = 67.11, SD 16.13) had lower grades than those with degrees or diplomas (mean = 80.32, SD 11.30) or some postsecondary experience (mean = 75.17, SD 12.22), *F* (2, 128) = 6.67, *p *=* *0.002. Post hoc Tukey test targeted that difference as being between high school entry and previous degrees for both paper (*p *=* *0.012) and final (*p *=* *0.001) percent grades. In addition, the findings neared significance between high school entry and some postsecondary education for final grades (*p *=* *0.053).

### 4.6 ESL status and past writing experience

As hypothesized, independent *t* tests showed no differences between students who self‐declared English as their second language and those who did not with respect to paper and final percent grades, SESAW, STAI and APA and grammar knowledge. A similar lack of significant difference was observed when the assessment compared those reporting less writing experience to those reporting more writing experience.

## 5 DISCUSSION

This study is unique in the body of literature examining WSE for several reasons. First, it examines WSE both pre and post a course with efficacy‐building scaffolded instructional methods and rules out the possibility of a maturation effect during a control time period where no academic writing was required. Second, it compares online and classroom instructional environments. Third, it examines WSE in self‐identified ESL and non‐ESL students, which has important implications for the instruction of writing in diverse student groups including international students. Fourth, it contributes valuable information for consideration when establishing admission policies, because nursing students direct out of high school struggle with the academic writing demands required to achieve passing grades.

As expected, WSE remained stable during the time control period when no writing was required and improved from pre‐ to postcourse in full study participants. In the experimental‐only group WSE improved but was not statistically significant. Failure to find significance in this latter group may have occurred for two possible reasons. First, the sample size was small with only 30 students in this portion of the analysis. Second, the initial WSE measure on this group was taken without a time control preceding their student experience. This group would have been responding to the first questionnaire, unaware of the nursing writing context and academic rigour of nursing education and this may have contributed to an over inflation of their initial self‐reported WSE giving little room for statistical improvement. Prat‐Sala and Redford (2012) made a similar observation in their first‐year psychology cohort. However, given that term one online students ultimately demonstrated a high degree of academic skill achieving the highest course final grades, their high WSE may have been justified.

Anxiety and WSE were negatively correlated as expected matching the findings of others when using writing apprehension or anxiety as the emotional arousal factor (Martinez et al., 2011; Mitchell et al., 2017; Pajares & Johnson, 1994; Sanders‐Reio et al., 2014). Surprisingly, anxiety did not change as a result of participating in the scholarly writing course. This finding is similar to the resilience in writing apprehension observed by Pajares and Johnson (1994). In this study, because anxiety was measured with the STAI, the STAI may have not been specific enough to writing anxiety and students may have answered the questions while envisioning their more general academic anxieties.

The APA/Grammar knowledge test produced some interesting trends in student knowledge of these writing tasks because participants demonstrated improvement of their knowledge during the control period (unexpected) as well as pre‐ to post course (expected). The improvement in scores from the beginning to the end of the control period was unexpected because students were not taking any writing instruction, completing formalized writing assignments, or being asked to apply APA style during the control phase. The noted improvement during the control period is more likely due to scoring the test by rewarding risk taking responses. By the end of their first term of study, students were more likely to guess at questions they were unsure of answers for—a trait related to learning effective test taking strategies. In addition, course readings in nursing may have exposed them to the patterns of APA in published textbooks and journals.

The most concerning but, perhaps, not surprising finding with respect to writing performance was that students who entered the programme without any previous postsecondary experience achieved the lowest writing course and paper grades while reporting similar WSE and anxiety at all measurement periods. Walsh, Prokos, and Bird (2014) noted that it is not unusual for inexperienced students to overestimate their capabilities in contexts where writing complexity and the demands of evaluators are unknown. This explanation also rationalizes the failure to find significance between students who self‐reported extensive past writing experience compared with those with little or remote past writing experience. Those who reported little writing experience likely had overestimated their WSE. Self‐evaluation is not likely to be accurate when little frame of reference is present for the experience. Context of writing is critical to accurate WSE measurements (Pajares & Johnson, 1994).

### 5.1 Online writing instruction

This study provides preliminary evidence that writing can be successfully taught in an online environment to generic baccalaureate nursing students. The online and classroom sections in second term only differed in terms of WSE at T1 with the online group reporting higher WSE. This difference is likely an indication that students heeded advice in section selection to choose the online version of the course only if they felt comfortable with their writing ability. Paradoxically, the second term online group ultimately achieved the lowest average final percent grades in the course indicating there may have been a mismatch between their self‐reported WSE and their writing ability. An overinflated sense of self‐efficacy in relation to grades, in some students, has also been observed by other authors (Williams & Takaku, 2011). The second term online students may have found themselves weaker in self‐regulatory skills to independently stay on pace with course materials (e.g. avoidance of the course in face of a heavy second term course load). However, because paper percentage grades did not differ significantly in this group when compared with the other sections, this is an indication that their final grades were lower as a result of poor quality of or failure to submit the other weekly assignments that contributed to their final grade. This pattern may be evidence that the second term online students did not attend to the course materials all that closely without having weekly class attendance to keep them accountable. By contrast, the first term online group had the highest final grades. This pattern may be an indication that student self‐selection of course section and previous college course credits prior to admission (required for admission to the first term online course) had a greater impact on grades in these online environments than WSE or anxiety levels. Some authors have suggested WSE instruments may be useful tools to guide course placement (Zimmerman & Bandura, 1994), however, if beginning students are prone to overinflate their sense of WSE, using the tool to place students in sections may not have the desired effect of appropriately grouping students by writing ability when disciplinary requirements and rigour are unknown to the student.

### 5.2 ESL status, WSE and writing performance

Identifying no differences between self‐declared ESL students and non‐ESL student in writing self‐efficacy, anxiety and grades was an important finding and similar to the results reported in Williams and Takaku (2011). Nursing instructors must be careful to not assume that ESL students are worse writers than their domestic counterparts, or overly scrutinize their work for errors because they are known ESL students.

### 5.3 Practical implications for teaching writing

#### 5.3.1 Scaffolding

Similar to an earlier study in this population (Mitchell et al., 2017), the results of this study contribute additional knowledge that scaffolding writing assignments in an introductory discipline‐specific writing course can enhance writing ability and WSE. Miller et al. (2015), in the only other published study examining WSE in nursing students, also used a scaffolding method of structuring their writing assignments. Their population was different from this study and from Mitchell et al. (2017) in that they explored nurse‐to‐degree students late in their nursing programme. Scaffolding combines two parallel processes: (i) structuring assignments so that they are completed in progressive stages over a term (Walsh et al., 2014) and, (ii) instructor involvement, where the instructor or the tutor act as the scaffold in the writing process, slowly withdrawing support as writers become more independent (Benko, 2013; Gazza & Hunker, 2012; Mackiewicz & Thompson, 2014).

Leveling assignments within a course and across a curriculum is achieved by increasing the complexity of written assignments. This approach requires the student to progressively demonstrate abilities in Bloom's taxonomy to first, summarize and describe and then synthesize, critically analyze and evaluate topics they are investigating (Gazza & Hunker, 2012). This process of reducing a major writing project into manageable relevant pieces may contribute to students being able to advance their thinking to higher levels of Bloom's taxonomy (Luthy et al., 2009). In the course described here, this process involved short writing assignments which began with reflection and then requested students summarize one source, followed by two more writing exercises where they were asked to synthesize two and then three sources on the same topic. Scaffolding also occurred in the scholarly paper assignment. The course prescripted a schedule which had students complete the major elements of the assignment in stages, first choosing their topic, searching for supporting research, learning to make notes from their sources and develop an outline and then writing and editing a rough draft in preparation for submission for grading (Mitchell et al., 2017).

#### 5.3.2 Instructor influence

While scholars who discuss writing instruction in nursing agree that writing is important to critical thinking skills, reasoning and career trajectories, we agree with Miller et al.'s (2015) conclusion that writing competency in many nursing programmes is assumed rather than taught. But because writing skills develop slowly, a single discipline‐specific course is not sufficient to develop proficient writers. All instructors who include a writing assignment as an evaluation criterion in a course must consider themselves writing instructors. Instructor involvement includes teaching course content in such a way that it connects the writing assignment to prior learning and future needs (Benko, 2013).

The scholarly writing course requires a tremendous number of instructor hours to implement successfully with large classes. Instructors must be comfortable with their knowledge of the writing process and their ability to give advice and feedback to students in a writing process framework for benefits to be observed. Scaffolding in courses is effective for any type of writing assignment but must include the critical elements of ensuring an appropriate challenge, allowing for student choice to increase engagement with writing and providing students with the opportunity to say something of their own, all contained in a caring classroom environment where instructors can show interest in student's writing ideas (Benko, 2013).

### 5.4 Study limitations

While this study successfully demonstrated that a discipline‐specific scholarly writing course using scaffolding as an instructional method can improve WSE in first year nursing students, the study does have some limitations. First, the original proposal for this study intended to compare this cohort of nursing students to another group of students in the college environment who were required to complete a significant academic writing assignment without the benefit of writing instruction, however, discussions with instructors in other programmes identified that all courses were providing too much writing support to function as a true control. While the time control period enhances confidence that the scholarly writing course positively affected WSE levels, a comparison group would have been an additional strength. Second, the bonus marks for participation were included in an attempt to curb attrition from the study, however, attrition remained high and was concentrated among students who achieved the lowest grades. Thus these results cannot be generalized to students who demonstrate poor writing performance.

### 5.5 Areas for future research

While this study successfully demonstrated that WSE can improve early in a nursing programme when assessed in the context of discipline‐specific writing, the empirical relationship between WSE and its role in student performance remains tenuous. In part, this is due to the difficulty in consistently assessing and scoring writing performance. The successful measurement of writing performance and detecting improvements in writing performance over time, requires similar writing assessment activities in a given course. For example, in this study, a second three‐page academic paper would have needed to be assigned early in the study—a requirement that would have put tremendous burden on both the students and the course instructors/graders. In addition, requiring a second intensive writing assignment early in a course prior to writing instruction would have likely produced high anxiety and some academically disastrous results for some students. One possible solution would be to explore the changes in drafts of the same assignment over a term.

This study used a brief grammar and APA test which was not to be considered an assessment of writing performance. It is generally well accepted among writing experts and theorists that good grammar (and application of APA format) does not equate to good writing as it is far too limiting a criterion to be a parallel assessment for performance. It may be argued that using a substitute writing assessment such as an in‐class essay is also not an adequate assessment of writing performance as it denies the student the opportunity to research, reflect, polish and be creative in their writing approach. These spontaneous essays are not a mirror for the kinds of assignments we require of students with the goal toward improving critical thinking, learning the discourse of a discipline, or creating a synthesis of knowing.

Miller et al. (2015) were likely correct in exploring changes in different sub‐components of writing performance (e.g. organization, sentence fluency, voice, or ideas), rather than exploring performance in a more global fashion such as grades or GPA, in their search for improvement over time. Williams and Takaku (2011) have suggested that the relationship between WSE and performance is likely mediated by student choices in help seeking for their writing. This study provides support for the idea that past writing experience (and more specifically, previous postsecondary experience) likely has a strong influence on writing performance in the form of grade earned in an assignment or course. But given that both experienced and inexperienced students reported similar WSE levels, this study also provided preliminary evidence that levels of WSE may bear little connection to past experiences of writing as WSE is not a stable construct and will fluctuate according to context, expectations and task requirements. Even expert writers will experience bouts of low self‐efficacy under conditions of changing expectations, challenging evaluators (sometimes external and sometimes self‐imposed) and disciplinary circumstances. However, improving writing self‐efficacy may play a role in shutting down negative self‐regulatory behaviours that lead to writing avoidance, stop students from writing, prevent them from making career choices that require writing such as advanced degrees, or keep them from writing to advocate for policy changes that may influence the nursing profession as a whole. Intervening in writing self‐efficacy could be what keeps students and nurses writing. These proposed relationships require further study.

The exploration of writing self‐efficacy and how it influences writing performance is a research area in its infancy. It is unlikely that writing self‐efficacy will dramatically improve performance across a single term as writing development is a lifelong endeavor. The only way writing performance can improve is through ongoing progressively more challenging writing. Currently a long‐term follow up of the same cohort of students is being conducted examining WSE and anxiety in terms of stability or growth over a curriculum. Help seeking, engagement with instructors, revision practices, response to feedback and progression through the programme will be investigated for their WSE connections. An exploration of the relationship between writing self‐efficacy and clinical practice success is being included. In addition to this active study, a revision of the WSE questionnaire is required to assess its discipline specific elements. Qualitative research examining the writing experiences of undergraduate nursing students are also necessary for establishing targeted interventions in this population.

## 6 CONCLUSION

This study has demonstrated that discipline‐specific writing instruction can influence writing self‐efficacy in first year nursing students. A paucity of research exists in nursing populations to address the “problem” of student writing. Scaffolding as an instructional method is a promising solution to improving student writing but requires intense instructor involvement from instructors who are confident with their own writing and confident assessing the writing of undergraduate students. Discipline‐specific writing, given that nurses communicate in a shared professional discourse, is the current recommended approach to improving writing and critical analysis skills in students at all levels of nursing education.

## CONFLICT OF INTEREST

No conflict of interest has been declared by the authors.

## AUTHOR CONTRIBUTIONS

Ms. Mitchell: designed, implemented and coordinated the study through all phases, developed and implemented the writing course, and wrote the manuscript.

Dr. Harrigan: contributed to the study design, provided statistical analysis and insights into the relevance of findings, and reviewed and approved the manuscript.

Dr. McMillan: provided insights to the statistical analysis, relevance of findings, and reviewed and approved the manuscript.
